# Reverse engineering gene regulatory networks: Coupling an optimization algorithm with a parameter identification technique

**DOI:** 10.1186/1471-2105-15-S15-S8

**Published:** 2014-12-03

**Authors:** Yu-Ting Hsiao, Wei-Po Lee

**Affiliations:** 1 Commerce Development Research Institute, Taipei, Taiwan; 2 Department of Information Management, National Sun Yat-sen University, 70, Lienhai Road, Kaohsiung, Taiwan

## Abstract

**Background:**

To infer gene regulatory networks from time series gene profiles, two important tasks that are related to biological systems must be undertaken. One task is to determine a valid network structure that has topological properties that can influence the network dynamics profoundly. The other task is to optimize the network parameters to minimize the accumulated discrepancy between the gene expression data and the values produced by the inferred network model. Though the above two tasks must be conducted simultaneously, most existing work addresses only one of the tasks.

**Results:**

We propose an iterative approach that couples parameter identification and parameter optimization techniques, to address the two tasks simultaneously during network inference. This approach first identifies the most influential parameters against internal perturbations; this identification is based on sensitivity measurements. Then, a hybrid GA-PSO optimization method infers parameters in accordance with their criticalities. The proposed approach has been applied to several datasets, including subsets of the SOS DNA repair system in *E. coli*, the Rat central nervous system (CNS), and the protein glycosylation system of yeast *S. cerevisiae*. The result and analysis show that our approach can infer solutions to satisfy both the requirements of network structure and network behavior.

**Conclusions:**

Network structure is an important though challenging issue to address in inferring sophisticated networks with biological details. In need of prior structural knowledge, we turn to measure parameter sensitivity instead to account for the network structure in an indirect way. By developing an integrated approach for considering both the network structure and behavior in the inference process, we can successfully infer critical gene interactions as well as valid time expression profiles.

## Background

Modeling gene regulatory networks (GRNs) is one of the most important issues in systems biology research. It uses time series gene profiles to characterize the phenotypic behavior of a target system, and reverse engineering has been advocated to construct networks in an automated way [1][2]. In the process of inferring gene networks, many computational models and methods have been proposed. The choices mainly depend on the biological levels to be studied and the computational complexities needed to solve the corresponding problems. To capture the sophisticated characteristics of a gene network, in this work we adopt one of the most popular and well-researched models, the S-system model, to represent a gene network. The S-system is a set of tightly coupled ordinary differential equations (ODEs), and the component processes in these equations are characterized by power law functions [3][4]. In the S-system model, the systematic structure can be described as:

(1) $$\frac{dX_{i}}{dt}=\underset{synthesis}{\underset{︸}{\alpha_{i}\prod_{j=1}^{N}X_{j}^{g_{i,j}}}}-\underset{degradation}{\underset{︸}{\beta_{i}\prod_{j=1}^{N}X_{j}^{h_{i,j}}}},\forall ;i$$

where *X_i _*is the expression level of gene *i *and *N *is the total number of genes in the genetic network. The parameters *α_i _*and *β_i _*∈ [0, 10] are rate constants (e.g., some constant input can be represented herein); *g_i,j _*and *h_i,j _*∈ [-3, 3] are kinetic orders that reflect the interactions from gene *j *to *i *in the synthesis and degradation processes, respectively. The inference of a tightly coupled S-system, however, is a large-scale parameter optimization problem that is very time-consuming. By examining the structural characteristics of gene networks, Maki *et al*. [5] proposed an efficient strategy to decouple this inference problem with *N *separated sub-problems, each of which refers to one gene. In other words, in a decoupled S-system, a tightly coupled system of non-linear differential equations is decomposed into *N *differential equations [6]. The main benefit of adopting this strategy is that it allows us to model corresponding genes and observe genetic interactions toward the target gene independently.

**Figure 1:**
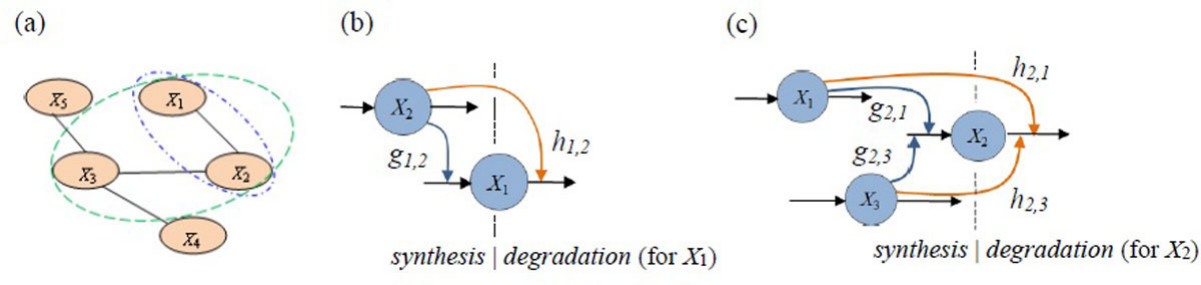
**Network topology and pathway diagrams**. (a) An example of a 5-node gene topology (unidirectional links); (b) and (c) are the pathway diagrams from the point of view of gene *X*_1 _and *X*_2_, respectively.

To exhibit how the components of an S-system represent a network topology and pathway diagrams, Figure 1a shows a visualized topology that was generated by an arbitrary arrangement. In the graph, we take gene *X*_1 _as an example, which has a link with *X*_2_; therefore, the pathway diagram can be expressed as in Figure 1b. The input magnitude (flux in) of *X*_1 _could be affected by *X*_2 _(i.e., *g*_1,2_), which is also called the synthesis process of *X*_1 _(the equation of *S_X1_*). At the same time, the output magnitude (flux out) of *X_1 _*depends on the concentration level of *X_1 _*(i.e., *h_1,1_*) and could be affected by *X_2 _*(i.e., *h_1,2_*) as well, which is depicted by the degradation process in the equation for *D_X1_*. The concentration of *X_1 _*at the next time step is determined by a calculation of the magnitude of synthesis minus that of degradation (i.e., *S_X1_*- *D_X1_*). The synthesis and degradation processes of gene *X_1 _*have the following relationships.

(2) $$\text{synthesis process}:\;S_{X1}=\alpha_{1}\;{X_{2}}^{g_{1,2}}$$

(3) $$\text{degradation process}:\;D_{X1}=\beta_{1}\;{X_{1}}^{h_{1,1}}{X_{2}}^{h_{1,2}}$$

(4) $$\overset{∙}{X_{1}}=S_{X1}-D_{X1}$$

Similarly, an example of the 3-node connections on the perspective of gene *X*_2 _(Figure 1c) but with the same computational process can be completed through the equations below.

(5) $$\text{synthesis process}:\;S_{X\text{2}}=\alpha_{2}\;{X_{1}}^{g_{2,1}}{X_{3}}^{g_{2,3}}$$

(6) $$\text{degradation process}:\;D_{X2}=\beta_{2}\;{X_{1}}^{h_{2,1}}{X_{2}}^{h_{2,2}}{X_{3}}^{h_{2,3}}$$

(7) $$\overset{∙}{X_{2}}=S_{X\text{2}}-D_{X\text{2}}$$

To infer target networks from time series gene profiles, many issues that are related to biological systems cannot simply be addressed by topology reconstruction or parameter estimation alone. As mentioned in [7], on the one hand, if the topology of a GRN can be reconstructed, it is usually not sufficient for a satisfactory scientific understanding (i.e., lacking the modeling of biological details). On the other hand, the optimized parameters for a given network topology (or mathematical structure) do not enable discrimination of alternative candidates. From the above analyses, we can recognize that there are two major goals to satisfy in inferring gene networks. The first goal is to minimize the accumulated discrepancy between the gene expression data recorded in the data set (desired values) and the values produced by the inferred model (actual values). The performance of a certain model can thus be defined directly as the mean squared error (MSE) over the time period:

(8) $$MSE\left(i\right)=\overset{T}{\underset{t=1}{\sum }}{\left\{\frac{X_{i}^{a}\left(t\right)\;-X_{i}^{o}\left(t\right)}{X_{i}^{o}\left(t\right)}\right\}}^{\text{2}}\text{, for }i\;=\;1,2,...,N$$

In the above equation, *X_i_^o^*(t) is the desired expression level of gene *i *at time *t, X_i_^a^ (t*) is the value generated from the inferred model, and *T *is the number of time points for measuring the gene expression data. *N *is the number of genes in the network. As mentioned previously, in this work, we adopt the decoupled strategy in which each sub-problem corresponds to the *i*-th MSE function.

The non-linear ODEs of an S-system are difficult to solve by traditional local optimization techniques [8][9], such as the conjugate gradient method and Newton's method. Global optimization techniques are better choices than the local techniques in estimating parameters for biological systems. Among the global methods, those utilizing deterministic strategies are more effective in finding the global optimum. However, they are computationally expensive. In contrast, the methods that employ stochastic strategies can obtain solutions close to the global optimum within a reasonable amount of time. Population-based approaches (such as genetic algorithms (GAs) or particle swarm optimization (PSO)) are stochastic methods, which have been used in many studies to infer S-system models (e.g., [4][10][11]). In this study, we adopt this type of global optimization technique and develop a new algorithm to enhance the search performance.

The second goal, in the meantime, is to select the solution that has a correct network structure. In real-world situations, the number of data points that are available is often smaller than that of the parameters to be determined; in other word, the network inference task is in fact an under-determined problem. It is thus possible to obtain many feasible solutions with various combinations of network parameters (i.e., different network structures). To solve the structure problem, prior knowledge or assumptions are required. Some researchers suggested that directly taking the form of a parameter constraint for the prior domain knowledge (extracted from the literature) would restrict the parameter search (for example, [11]). Other researchers proposed to incorporate the structural/topological properties of the biological networks (such as the degree distribution of the nodes in a scale-free network or the presence of network motifs that have been interpreted to be the result of evolutionary dynamics) with the gene expression data described above in the evaluation process. For example, some inference methods intend to limit the amount of GRN connectivity to be as small as possible because gene regulatory networks are typically known to be sparsely connected (i.e., every gene interacts directly only with a few other genes). In such a case, a small penalty term that measures the connection between the genes can be added to the fitness function to discourage the connections [10][12]. To take both gene expression data and structure information into consideration, the evaluation function for network inference thus becomes:

(9) $$f_{obj}\left(i\right)=\alpha \cdot MSE\left(i\right)+\left(1-\alpha \right)\cdot StructureErr,\text{ for }i=\text{1}\sim N$$

where MSE is the mean squared error, StructureErr is a penalty that describes the structure discrepancy between the current solution and the previously known knowledge, and α is a weighting factor between 0 and 1.

Prior knowledge, however, is not always available. Additionally, the topological properties of the network that are unveiled within the genome-scale networks cannot be easily addressed in studies of relatively small size networks (that were often used in sophisticated network modeling, to better understand their biological details). Though some tools can be used to derive skeletal network structures from time-series data, for example BoolNet [13], their results are not sufficiently accurate to be used as structural knowledge to guide the search for solutions. In this study, we use a different perspective when accounting for the network structure in the inference procedure. Because several theoretical analyses have demonstrated that a gene regulatory network with correct structure innately has the intrinsic characteristic of *robustness *(e.g., [14][15]), we instead turn to infer robust networks that are likely to have the correct structures when there is a lack of explicit structural knowledge.

Network robustness can be defined as the insensitivity of a specific system property to variations in the components and environment of the system [16][17]. In general, there are three types of robustness against different types of perturbations that are often considered in the literature: knockout robustness, parametric robustness, and initial condition robustness [18]. The first two types of robustness are related to the evolutionary (genetic) perturbation (i.e., mutations that cause a gene or protein to be non-functional and that effect the binding strength of the transcription factors to their targets, respectively), and the third type of robustness involves the environmental perturbations (i.e., environmental shifts that affect the concentrations of various proteins, nutrients, and gene transcripts). Some prior studies have shown that there is a growing consensus that the network structure has a significant influence on the robustness [18][19]. Though robustness is a critical feature for living systems, details about the mechanisms through which robustness is achieved are still not well understood.

As mentioned above, we focus here on how to infer a network that is robust against internal fluctuations caused by the parameters that correspond to active gene interactions. In a biological system, genes interact within a complex network to provide robust functions, and each network parameter has its role in determining the system behavior. Nevertheless, several authors have observed that most of the variation in the measured gene expression changes can be explained by a relatively small number of variables [20][21][22]. Parameters that correspond to these variables are very sensitive to variations and can introduce fragility into the system. To ensure the robustness of the inferred network and to further investigate gene interactions, it is thus important to first identify the critical subset of the network parameters based on the observed system changes and, then, to derive an acceptable value range for each parameter to restrict its value lying in the specified range during the network reconstruction process [17]. Therefore, we take parameter sensitivity into consideration in the network reconstruction procedure in such a way that robust results (i.e., insensitivity to internal variations) can be obtained.

In this work, we present an integrated approach, which is revised from our previous study, which focused on parameter sensitivity analysis (SA, [23]) to iteratively evolve partial solutions to guide the search gradually toward the complete solution. This approach couples *parameter identification *and *parameter optimization *techniques to address the aforementioned two problems in gene network inference: it first identifies which parameters are more critical than others in a system, based on their sensitivity measurements. As mentioned before, this arrangement is to enhance the structure correctness of the inferred network in an indirect way, under the situation that lacks prior knowledge about the network structure. Then, it involves a hybrid GA-PSO optimization method to infer networks in accordance with the parameter criticality. To validate the proposed approach, a series of experiments have been conducted on artificial and real datasets. The results and analysis show that our approach can infer robust networks with desired system behavior successfully from the gene profiles.

## Methods

**Figure 2:**
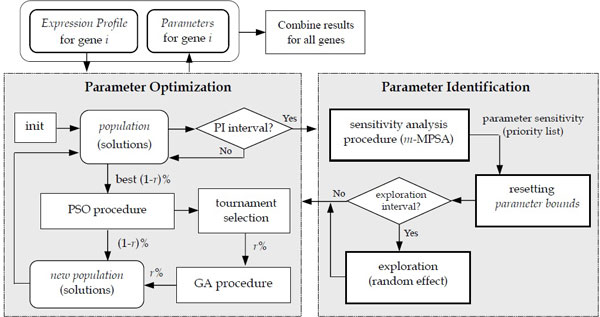
**Main flow of the proposed approach**. The proposed approach includes two major procedures: the left-hand portion is an evolutionary mechanism that was developed for parameter optimization, and the right-hand portion is the parameter identification procedure that was developed to work with the optimization procedure.

Figure 2 depicts the main flow of the proposed approach, which includes two procedures for parameter identification and parameter optimization, respectively. The left-hand portion shows an evolutionary mechanism that was developed for parameter optimization. Because recent surveys of population-based algorithms have revealed that the hybrid methods of GA and PSO can lead to better results in solving optimization problems than the individual methods alone, we therefore extend the hybrid method that we developed previously ([11]) to address the parameter optimization. The right-hand portion of Figure 2 depicts the parameter identification procedure that was developed to work with the optimization procedure. This procedure is performed when the evolution proceeds to a predefined number of iterations: it mainly includes a sensitivity analysis method for calculating the parameter sensitivity, selecting the most sensitive network parameters and determining the value ranges for them (i.e., to work as implicit structural knowledge), and then, it sends the parameters with the constraints back to the optimization procedure to continue the search. The above two phases operate in an iterative manner to keep the MSE and sensitivity of the network low. Once the parameters for all of the genes are determined, they are combined to constitute a network. The details are described below.

### Parameter identification

As mentioned above, in an inference method, it is important to identify the critical network parameters (with a certain level of significance) based on the observed system changes before performing parameter estimation because the parameter values are only valid for the set of parameters that were selected (which imply a specific/correct model structure). The parameter identification procedure employed here is based on a sensitivity analysis technique, in which the parameter values are adjusted within a given range and then the statistical calculations are employed to measure the system stability. With the parameter sensitivity measurement defined in [24], we can identify the critical parameters (which correspond to the active interactions that govern the dynamics of the system) or discover parameters that could influence the synthesis and the degradation processes of a network.

Two types of approaches are often utilized to implement the SA techniques for a dynamic system, namely local SA and global SA. Local methods focus on a specific point in parameter space and measure responses of the model to local parameter changes. The advantage of this type of approaches is that when the network structure remains unknown, local SA methods still can give the sensitivity estimation by calculating a parameter's given value range. Local SA methods, however, consider only one parameter per calculation and do not account for other parameters (interactions) [25]. This approach often results in an underestimation of the true model sensitivities in non-linear models. In contrast, global methods attempt to capture the entire parameter space. They explore multiple parameter values and examine parameter interactions with different parameter values simultaneously [26]. However, this type of methods can only be used if the network structure is known beforehand.

In the case of inferring a gene network from the expression data, the network structure is usually unclear during the modeling process. It is therefore impossible to locate and choose the most important genetic interactions as in the analysis pool for global SA. To consider multiple network parameters simultaneously, we devise a new approach modified from a widely used SA technique, which is multi-parameter sensitivity analysis (MPSA, [24]). MPSA utilizes *Pearson correlation coefficients (PCC*) for quantitative comparisons to identify the sensitive parameters during inferring a GRN, which is achieved by calculating and ranking the values of PCC among parameters. This approach can be used to explore influential genetic interactions and to examine the robustness of an inferred GRN, similar to other global SA methods. Our newly developed method (called *m*-MPSA) includes an iterative process for calculating the sensitivity of each parameter, and then ranks the sensitivities of all of the parameters. By considering the most sensitive parameters first, the inference algorithm can then obtain robust solutions.

In the parameter identification procedure, a parameter range *R_i _*is set for each parameter *i*, and a set of random points (500 in this study, determined by a uniform distribution as suggested in [24]), are created within a specified interval. Each random point (value), together with the other parameter values, constitutes a candidate solution, and its fitness value is calculated via the objective function. A pre-defined threshold *C_r _*(defined as the triple of the best fitness value among all individuals, based on a preliminary test) is used to determine whether the fitness value of each random point is acceptable or unacceptable. Then, the sensitivity for each parameter *i *(defined as sensitivity*_i_*) is calculated using its cumulative frequency (*CF_i_*), which measures the *correlation coefficient *(i.e., PCC) of the acceptable *CF_i _*and the unacceptable *CF_i _*values. Finally, the parameters are ranked based on their sensitivity values. The parameters with relatively low sensitivity values are considered to be sensitive (influential). The additional details of how the cumulative frequency, and sensitivity of a parameter are calculated are given in our previous work [23].

### Parameter optimization

In addition to the above parameter identification procedure, the hybrid GA-PSO procedure for parameter optimization also plays an important role in the proposed approach. In this approach, a direct encoding way for designing an individual of the population is adopted. The parameters in the S-system for gene*_i _*(i.e., *α_i_, β_i_, g_i,j_*, and *h_i,j_*) are floating-point numbers and arranged linearly. To evaluate the performance of each potential solution (i.e. the combination of network parameters), a fitness function is defined to be the error function described in the first section (i.e., equation (8)) of the performance measurement.

The optimization procedure operates in the following way. The GA-PSO procedure first generates a random population (containing *n *individuals) and evaluates these individuals (to determine their fitness values). Then, the individuals are ranked based on their fitness values and separated into two parts: (1-*r*)% and *r*%, where (1-*r*)% is the best part of the population. After that, the GA and PSO processes are performed as follows. First, the (1-*r*)% individuals are preserved and enhanced by the PSO procedure. In the meantime, the *r*% individuals (i.e., the ones not selected) are pending, awaiting updates by the GA procedure. Second, to replace those pending individuals, new individuals are selected through a tournament selection scheme, and then sent for the GA operations (e.g. crossover). The newly generated individuals fill in the pending part of population (i.e., the *r*% mentioned above). Finally, once a new candidate list is formed, the individuals in this list are again ranked based on their fitness values, and the new population is dispatched to the next generation. The above procedure is repeated until the termination criterion is met. For simplicity, in this work, the randomness rate *r *is set to 0.5, though it could be a variable with a value that changes during the run to control the population diversity (i.e., to coordinate the progress of the PSO and GA parts).

The main steps of the integrated approach are described in Algorithm PIO, in which Step 2 indicates the evolution cycle for deriving the network parameters. As seen in Step 2, there are two strategies used in the evolution. The first strategy (i.e., Step 3) is to perform sensitivity analysis by the *m*-MPSA method described above, and then a threshold *CCR *(i.e., the correlation coefficient ratio of the *CF *values, or sensitivity*_i_*, obtained from the sensitivity analysis procedure) is adopted as a selection baseline for determining the most sensitive parameters. After the parameters have been classified, new value ranges (i.e., *R_i _*in *m*-MPSA) are set for the parameters: the sensitive parameters are given tight intervals, whereas the insensitive parameters are assigned loose intervals. The second strategy is an exploration phase (i.e., Step 4) to maintain population diversity and to avoid having individuals move close to locally optimal solutions. Currently, the above two strategies are performed periodically (the parameter identification occurs every 500 generations, and the exploration interval occurs every 1000 generations).

In this algorithm, the most critical strategy for achieving the network inference lies in Step 3. In detail, this strategy is intended to perform sensitivity analysis for the network parameters and to specify the constraints (value ranges) on them. A network parameter (i.e., a search dimension) is added to the sensitive list and is given a pair of tight constraints (specified by the upper and lower bounds, in which *α *is 2 and *β *is 5, based on a preliminary test) if its sensitivity value is less than (or equal to) the threshold *CCR*; otherwise, a parameter is added to the insensitive group and assigned a pair of loose constraints. The dimensions corresponding to the parameters recorded in the sensitive list are given a higher priority with respect to being searched, and their parameter values must be determined at an earlier evolutionary stage. As described in Step 3, an operation of classification is performed to group sensitive and insensitive parameters. The search space for each parameter is set to either a small-range interval or a relative large interval, according to the classification result. With a specified interval, the optimization procedure is performed to derive suitable parameter values. In algorithm PIO, the interval is equal to a search constraint associated to a parameter. Nevertheless, the constraint can be regarded as search guidance in finding the sensitive (influential) parameters. It can help a non-deterministic search method (e.g. an evolutionary algorithm) concentrate on exploring the pre-defined space (i.e. the specified intervals) and rule out solutions with feasible but fragile values. It means that if a parameter value goes behind the designated interval, the system dynamics will be affected severely and that will change the network behavior consequently. Hence, setting and observing the intervals (especially for the sensitive group) during the search process are helpful in acquiring robust networks with desired behaviors. In addition, as pointed out in [16][17], by understanding how the parameters vary according to different bounds towards different system dynamics, scientists may have new insights into the underlying mechanism corresponding to the observed biological behaviors.

As seen in Step 3, the threshold *CCR *will be increased gradually (by a small amount *ε*, which is equal to 0.01), and the parameters on the insensitive group will be turned into the sensitive group incrementally. In this way, parameters will be recognized as sensitive eventually, and their values will thus be constrained to a limited range. Because the sensitive parameters are more influential to the network behavior, enforcing restrictions (which are derived from the best available solution) to these parameters remains the robustness of the whole gene network and, meanwhile, evolves other parameters during the optimization process. Finally, the proposed algorithm can lead the inferred solutions to meet both the desired network robustness and system behavior.

Other details about the variables used in Steps 3 and 4 are described as follows. *p[gbest*] is the individual (solution) with the best fitness value over the entire population (i.e., the globally best in the PSO), and *p[gbest_i_]* represents the *i*-th parameter of *p[gbest]*. In addition, *p[particle_j_]*[*LocalBest]* is the individual with the best fitness value obtained from the flying history of the *j*-th particle (i.e., the locally best in the PSO), and *p[particle_j_]*[*LocalBest_i_]* represents the *i*-th parameter value of *p[particle_j_]*[*LocalBest]*.

**Algorithm PIO: **Integrated Approach of Parameter Identification and Optimization

1   Input: a gene expression profile (time-series data)

2   Output: values of the network parameters

3   **Step 1: **Initial setting.

4     Setting the experimental parameters P: the generation number *max_gen*, the correlation coefficient ratio

5     *CCR*, and the PSO parameter *velocity_bound*.

6   **Step 2: **Running the main evolution-cycle, as follows:

7     **for **(*gen *= 1 to *max_gen*)

8       **if **(parameter identification interval) **then**

9         Go to Step 3**;**

10         **if **(exploration interval) then

11           Go to Step 4;

12       Infer S-system parameters by running the GA-PSO procedure;

13     **end_for**;

14   **Step 3**: (parameter identification phase)

15     **call ***m*-MPSA for sensitivity analysis;

16     Use a threshold *CCR *to classify the parameters as *sensitive *or *insensitive*;

17     Sort the particles in ascending order according to their fitness values;

18     **for **each particle *j */* Set new value ranges for all of the network parameters

19       Give tight bounds for each *sensitive *parameter *i*:

20         upper bound *UB_i _= p[gbest_i_*] + *α *× *velocity_bound*;

21         lower bound *LB_i _= p[gbest_i_*] - *α *× *velocity_bound*;

22       Regenerate its value according to new *UB_i _*and *LB_i_*;

23       Give loose bounds for each *insensitive *parameter *i:*

24         upper bound *UB_i _= p[gbest_i_*] + *β*×*velocity_bound*;

25         lower bound *LB_i _= p[gbest_i_*] - *β*×*velocity_bound*;

26       Regenerate its value according to new *UB_i _*and *LB_i_*;

27       Let *p[particle_j_*][*LocalBest_i_*] equal to the regenerated value;

28         Update *p[gbest*], if *p[particle_j_*][*LocalBest_i_*] performs better than *p[gbest*];

29     **end_for;**

30       *CCR = CCR + ε*; /* update the threshold to consider more parameters in the next parameter

31                           identification interval

32     return;

33   **Step 4: (**exploration phase)

34     **for **each particle *j *(except for the top 1% of the particles)

35       **for **each network parameter *i*

36         Regenerate its value according to new *UB_i _*and *LB_i_*;

37         Regenerate the velocity of the particle swarm;

38         Let *p[particle_j_*][*LocalBest*] equal to the regenerated value;

39       **end**_**for;**

40       Update *p[gbest*], if *p[particle_j_*][*LocalBest*] performs better than *p[gbest*];

41     **end_for;**

42     return;

## Results and discussion

In this section, we describe how we conducted a series of experiments to verify the developed integrated approach from two different perspectives: the external network behavior and the internal network robustness. In these experiments, we first examined the performance of applying computational methods with parameter sensitivity analysis to two artificial datasets, and then, we focused on the evaluation and analysis for three real-world datasets.

### Evaluation of the proposed approach on artificial datasets

In the first set of experiments, several artificial datasets often used in gene network inference were collected to evaluate the proposed approach. Because our major goal is to investigate how the proposed approach can be adopted to infer networks for real datasets (see the experimental analysis in a later section), we report here only two sets of the results as representative examples. The first dataset is a five-node network taken from [27], in which the nodes and their interactions are described by the following non-linear relationships:

$$\begin{array}{c} {Ẋ}_{1}=15.0X_{3}X_{5}^{-0.1}-10.0X_{1}^{2.0}\\ {Ẋ}_{2}=10.0X_{1}^{2.0}-10.0X_{2}^{2.0}\\ {Ẋ}_{3}=10.0X_{2}^{-0.1}-10.0X_{2}^{-0.1}X_{3}^{2.0}\\ {Ẋ}_{4}=8.0X_{1}^{2.0}X_{5}^{-0.1}-10.0X_{4}^{2.0}\\ {Ẋ}_{5}=10.0X_{4}^{2.0}-10.0X_{5}^{2.0} \end{array}$$

The second dataset is a ten-node network taken from [10], in which the nodes have the following non-linear relationships:

$$\begin{array}{c} {Ẋ}_{1}=5.0X_{4}X_{6}^{-2.0}-10.0X_{1}^{2.0}\\ {Ẋ}_{2}=10.0X_{3}X_{8}^{1.0}-10.0X_{2}^{2.0}\\ {Ẋ}_{3}=8.0X_{1}^{-1.0}X_{4}^{-1.0}-10.0X_{3}^{2.0}\\ {Ẋ}_{4}=10.0X_{5}^{2.0}X_{9}-10.0X_{4}^{2.0}\\ {Ẋ}_{5}=10.0X_{2}^{2.0}X_{6}^{-1.0}-10.0X_{5}^{2.0}\\ {Ẋ}_{6}=5.0X_{9}^{2.0}X_{10}^{-2.0}-10.0X_{6}^{2.0}\\ {Ẋ}_{7}=10.0X_{6}X_{10}^{-1.0}-10.0X_{7}^{2.0}\\ {Ẋ}_{8}=5.0X_{1}X_{2}^{-2.0}X_{7}-10.0X_{8}^{2.0}\\ {Ẋ}_{9}=10.0X_{3}X_{8}^{-2.0}-10.0X_{9}^{2.0}\\ {Ẋ}_{10}=8.0X_{1}^{2.0}X_{7}^{-1.0}-10.0X_{10}^{2.0} \end{array}$$

To collect gene expression data, we started and continued network operations for thirty simulation steps. Before using the developed approach to infer robust results, we conducted an investigation on the above two datasets to examine the effect of considering the network structure in the inference procedure. In this set of experiments, to prioritize the network topology of the inferred model toward the ideal structure, a structural correctness function was added to the MSE function given in the first section (i.e., equation (8)). This evaluation function includes two major parts, as follows:

(12) $$f_{obj}\left(i\right)=\alpha \cdot MSE\left(i\right)+\left(1-\alpha \right)\cdot \frac{StrPri\left(i\right)}{2N},\text{ for }i=\text{ 1, 2, 3, }...\text{, }N$$

The first function, *MSE(i*), is used to derive the correct network behavior, whereas the second function, *StrPri(i*), is used to minimize the structure inconsistency between the structure suggested by the prior knowledge and that of the inferred model. The weighting factor *α *is used for controlling the importance of the two issues to be considered (i.e., the network behavior and structure). There are two sub-terms for the structural priority function as described below.

(13) $$sp_{1}\left(i\right)=\frac{nzp_{i}}{zPK_{i}}\text{; }sp_{2}\left(i\right)=\frac{zp_{i}}{nzPK_{i}},\text{ where }sp_{1}\left(i\right)=\frac{nzp_{i}}{zPK_{i}}\text{; }sp_{2}\left(i\right)=\frac{zp_{i}}{nzPK_{i}}$$

In this equation, *sp_1 _*∈ [0, 1] is the ratio of the parameters against the suggestions from prior knowledge: there is no plausible connection between gene *j *and *i*. In detail, the numerator *nzp_i _*∈ N^0 ^(i.e., non-negative integer) records how many inferred kinetic orders of gene *i *are non-zero (i.e., meaning the connections) but should be zero according to prior knowledge. The denominator *zPK_i _*represents the total number of kinetic orders of gene *i *that are zero, (i.e., there is no link to gene *i *given by prior knowledge). In contrast, *sp_2 _*∈ [0, 1] is also the ratio of parameters, but it indicates that the kinetic orders of gene *i *follow the suggestion that a plausible connection should exist between genes *j *and *i*. Hence, the numerator *zp_i _*∈ N^0 ^counts the number of zero values from the inferred kinetic orders, and the denominator *nzPK_i _*represents the total number of kinetic orders of gene *i *that are non-zero.

Once the evaluation function was defined, we conducted thirty independent runs for each dataset with a population size 200, in which each run lasted for 500 iterations. Tables 1 and 2 show the results (averaged over thirty runs) of using a different weighting ratio *α *for the two datasets. As observed, when the structure error was introduced to the evaluation function, the resulting model tended to have a structure that was closer to the original network but that had a less-fitted behavior. These results show the importance of the network structure and indicate the need for an inference approach to account for both issues (i.e., the network behavior and the structure).

**Table 1:** Effect of considering structure correctness in the evaluation function for dataset 1.

behavior: structure	(GA-PSO)	1:9	3:7	5:5	7:3	9:1
*fitness *(avg)	1.15E-03	5.16E-03	2.12E-03	1.13E-03	1.40E-03	1.33E-03
*structure *(avg)	33.87%	86.60%	80.13%	75.07%	69.53%	61.40%

**Table 2:** Effect of considering structure correctness in the evaluation function for dataset 2.

behavior: structure	(GA-PSO)	1:9	3:7	5:5	7:3	9:1
*fitness *(avg)	1.33E-02	4.10E-02	2.44E-02	2.06E-02	2.49E-02	1.41E-02
*structure *(avg)	24.85%	70.80%	59.57%	53.88%	45.78%	37.23%

After presenting the influence of the network structure, we performed experiments to evaluate our approach that considers network robustness as a core factor for representing the unknown network structure. For the above two datasets, two optimization algorithms (i.e., the traditional PSO method and our hybrid GA-PSO method) with different settings (without and with sensitivity analysis for network parameters) were arranged, and twenty independent runs were conducted for each arrangement. Table 3 shows the results, in which the mean, standard deviation, and best and worst performance of the runs are listed for each arrangement (without, indicated as w/o, and with sensitivity analysis).

**Table 3:** Fitness and sensitivity obtained from different settings.

	PSO				GA-PSO			
	
	dataset 1		dataset 2		dataset 1		dataset 2	
	w/o	with	w/o	with	w/o	with	w/o	with
Avg	2.02E-03	1.17E-03	8.69E-02	4.62E-02	4.34E-04	1.45E-04	3.04E-02	1.23E-02
Best	2.97E-04	5.79E-04	1.76E-02	1.39E-02	2.47E-04	4.90E-05	2.42E-03	6.66E-04
Worst	5.50E-03	2.72E-03	2.46E-01	1.42E-01	1.57E-03	4.62E-04	9.51E-02	5.76E-02
S.D.	1.79E-03	5.48E-04	6.42E-02	2.98E-02	2.45E-04	9.56E-05	3.29E-02	1.70E-02
Sensitivity	0.7703	0.7281	0.6929	0.6468	0.7844	0.7388	0.7684	0.7462

From Table 3 we can observe that both of the computational methods using SA consistently outperform the other methods that do not use SA in network inference, and the proposed GA-PSO method performed better than the traditional PSO method in all runs. This table indicates that the proposed approach obtained better results on the average, best, and worst fitness values, and it provided smaller standard deviations. To evaluate the robustness of the evolved gene networks, we also list the sensitivity values (averaged over all parameters) of the best solutions obtained from the final generations for two settings. They show that the runs with SA could evolve networks that had lower fitness values (i.e., better system behavior) and lower sensitivity values (i.e., more robust networks) simultaneously. It should be noted that the threshold *CCR *is a relative value that is determined by the statistical distribution of all parameters, and it has a different value for each algorithm. Therefore, it is not suitable for comparing the sensitivity values (which rely on the thresholds) of different algorithms.

**Figure 3:**
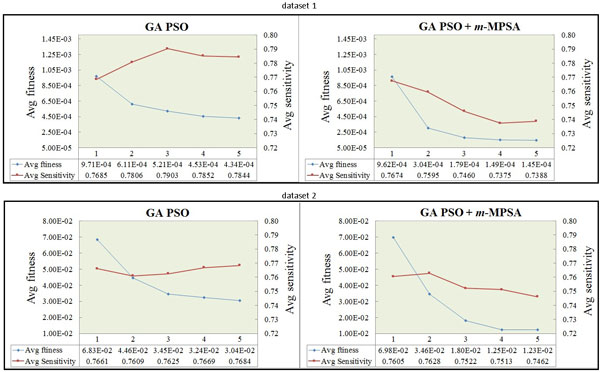
**Variations of fitness and sensitivity during the runs**. The fitness (left *y*-axis) and sensitivity (right *y*-axis) of the runs without and with sensitivity analysis for two the datasets. Each unit of the *x*-axis represents 500 generations.

Figure 3 illustrates how the fitness and sensitivity values (averaged over twenty runs) varied during the inference process by the GA-PSO without (left) and with (right) SA. This figure indicates that in the runs with our specially designed procedure for sensitivity analysis and parameter selection both the fitness and sensitivity curves went down stably after a certain number of iterations. These results confirm the efficiency and effectiveness of the proposed approach.

### Importance of parameter sensitivity analysis: studies of real systems

After evaluating the performance of our integrated approach for network inference, we conducted a set of experiments to investigate how our approach could be applied to the study of real gene networks. As the GA-PSO algorithm has been shown to outperform the other method, in this set of experiments, we chose to use this method with two settings (with and without SA) to conduct experiments on three popular real-world datasets.

**Figure 4:**
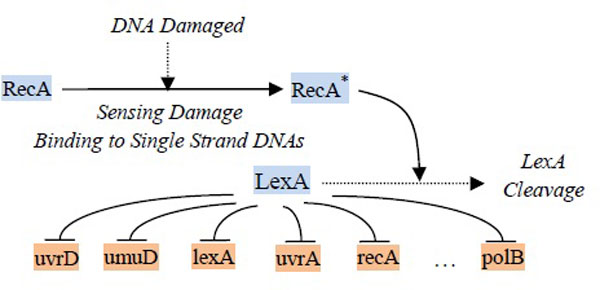
**SOS DNA repair system in *E.coli***. The gene regulations within the SOS DNA repair system in *E.coli*.

The first dataset is the SOS DNA repair system in *E. coli*. Figure 4 illustrates how the gene regulations response to DNA damage in the cell [28]. This system, on the one hand, involves the repressor LexA, which keeps activating and therefore represses the SOS genes (lexA, polB, recA, umuD, uvrA, and uvrD). On the other hand, the RexA protein involves the inactivation of the LexA in this system. When RecA is activated (it senses DNA damage), it interacts with LexA, which causes the expression level of LexA decrease (the repression becomes weak), and the concentration of the SOS genes thus rises. The fully expressed SOS genes then activate the repairing process. Once the damage has been repaired, the concentration of recA drops and this stops facilitating the self-cleavage of the LexA repressor. The expression level of LexA increases and begins to repress the SOS genes again. Recently, several studies have uncovered the regulations of the six SOS genes mentioned above, and the network structure has been reconstructed (e.g., [29][30]). We therefore chose these six genes from the original experimental data reported in [31] for our study here. Using the regulatory relationships described, we can validate our proposed method by comparing the results to those reported previously.

**Figure 5:**
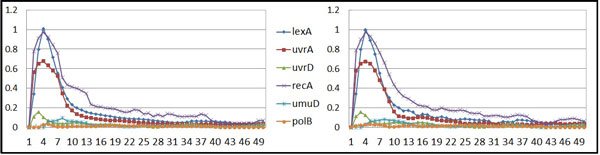
**Inferred network behavior**. The inferred (left) and original (right) network behaviors for the SOS dataset.

The SOS repair system dataset includes 50 time points. In this experiment, the decoupled S-system model was used and twenty runs were performed with two settings: without and with SA techniques. The results are presented in Table 4 in which the average fitness and the average parameter sensitivity values are revealed. These results indicate that the approach coupled with a sensitivity analysis procedure outperformed the algorithm without using SA (better fitting curves and lower sensitivity values can be derived). Furthermore, Figure 5 compares the inferred and target network behaviors (the *x *and *y *axes represent time steps and the concentrations of specific gene products, respectively), in which the inferred expression profiles are very close to that of the real system.

**Table 4:** Results of the SOS dataset by methods with and without parameter sensitivity analysis.

	lexA		uvrA		uvrD	
	w/o	with	w/o	with	w/o	with
	
*fitness*	1.5778	0.5360	1.8404	0.7447	4.0382	1.3453
*sensitivity*	0.8355	0.7977	0.8417	0.7838	0.7890	0.7890
	**recA**		**umuD**		**polB**	
	w/o	with	w/o	with	w/o	with
	
*fitness*	3.0527	1.8589	6.0470	4.1333	21.1221	14.6093
*sensitivity*	0.8669	0.7943	0.8315	0.7841	0.8507	0.7787

**Figure 6:**
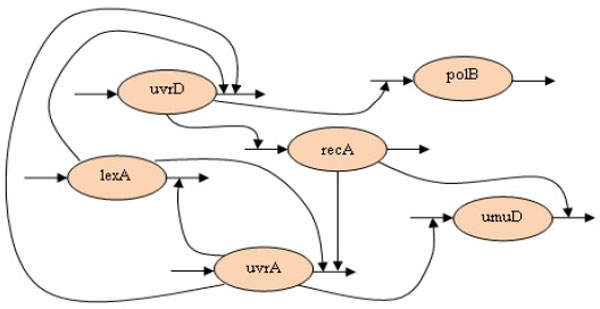
**Pathway diagram of the most crucial parameters**. An overview pathway diagram. The most sensitive parameters of the 6-gene SOS repair system identified by the proposed approach as shown in Table 5.

To investigate the parameters that have significant effects on the system dynamics of the SOS repair system, we summarized the most sensitive parameters identified by the proposed PIO algorithm. Thirteen parameters were highlighted as crucial regulations, each of which represented a regulatory relationship between two genes. Table 5 lists the gene regulations that were also found in the literature. Based on this table, Figure 6 depicts the pathway diagram of the most crucial parameters (genetic interactions) on both the synthesis and degradation processes of each gene. The results show that, of the thirteen gene regulations found in our experiments, ten matched regulatory relationships known from other studies. For example, the regulation of lexA, uvrA, and uvrD by lexA degradation has been successfully identified as the most significant regulations. However, the regulation of lexA by recA degradation was not marked by the PIO algorithm. This is because our approach operated on the gene level, whereas the regulation between lexA and recA was affected by LexA and RecA at the protein level [29]. In detail, the representation of the S-system model had inherent limitations on discovering genetic interactions that involved biological details in both protein and gene levels. These limitations were not related to the inference algorithm, therefore, the corresponding problems were not addressed in the inference process. Nevertheless, the proposed approach has shown its advantages that can capture the expected network behavior and determine the network robustness in modeling a real gene system.

**Table 5:** Critical gene regulatory relationships in the SOS repair system (→ means the synthesis process and -| means the degradation process).

gene	gene regulation
**lexA**	lexA -| lexA, lexA -|uvrA, lexA -| uvrD
**uvrA**	uvrA -| lexA, uvrA -| uvrD, uvrA → umuD
**uvrD**	uvrD → recA, uvrD → polB
**recA**	recA -| uvrA, recA -| umuD

In addition to the above SOS dataset, we also applied the proposed approach to the second real experimental dataset for further verification. This dataset is from the Rat central nervous system (CNS) taken from [32], which included expression data of 112 genes collected from 9 time points of different phases (embryonic, postnatal, and adult). The gene expression profiles used here are in the cluster with 17 genes derived from the Rat CNS dataset. Table 6 lists the genes. Twenty runs were conducted, in which 612 parameters in the network model must be analyzed during the evolution.

**Table 6:** The gene set and the number of sensitive parameters (regulations) activated by each gene in the synthesis or degradation process.

Gene	bFGF	NEFH	aFGF	mAChR4	S100 beta	GRG1	GFAP	IP32R	ChAT
*Regulations*	10	9	8	6	6	6	5	5	4
Gene	mGluR1	NFM	c-fos	c-jun	MOG	CNTF	NGF	NMDA2A	
*Regulations*	3	3	2	2	2	2	1	0	

**Figure 7:**
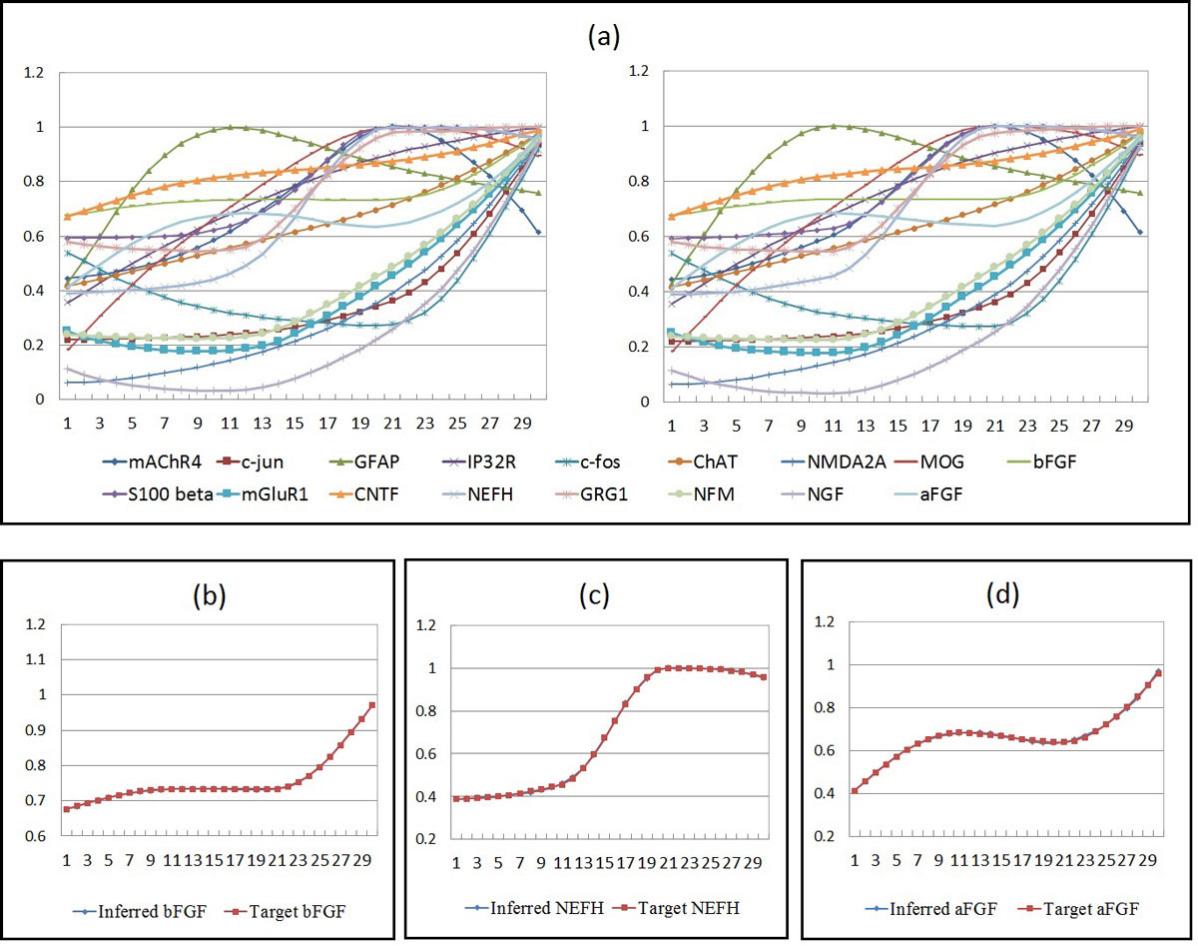
**Analysis of the inferred network behavior**. (a) An overview of the inferred (left) and original (right) network behaviors of the rat CNS dataset. The target and inferred expression profiles of the bFGF, NEFH, and aFGF genes are given in (b), (c), and (d), respectively.

In the experiments, the hybrid GA-PSO method was employed to construct the CNS network. Two sets of experimental runs (without and with sensitivity analysis) were conducted, as in the previous sections, and the results are presented in Table 7. As observed, the optimization method with sensitivity analysis performs better than the other case on both the fitness and sensitivity. In addition, Figure 7 depicts the system behavior (i.e., the time series data) of the original and the inferred networks, in which the *x*-axis shows the time steps and the *y*-axis shows the expression levels of the genes. Again, the figure shows that the inferred network can produce very similar behavior to that of the original network.

**Table 7:** Results of the CNS dataset from different strategies.

	mAChR4		c-jun		GFAP		IP32R		c-fos		ChAT	
	w/o	with	w/o	with	w/o	with	w/o	with	w/o	with	w/o	with
*Fitness*	0.0014	0.0006	0.0006	0.0001	0.0004	0.0001	0.0008	0.0003	0.0016	0.0004	0.0009	0.0003
*Sensitivity*	0.7828	0.7595	0.7262	0.7019	0.7494	0.7149	0.7358	0.7153	0.7877	0.7395	0.7697	0.7383

	**NMDA2A**		**MOG**		**bFGF**		**S100 beta**		**mGluR1**		**CNTF**	
	w/o	with	w/o	with	w/o	with	w/o	with	w/o	with	w/o	with

*Fitness* *sensitivity*	0.0008	0.0002	0.0007	0.0002	0.0028	0.0001	0.0012	0.0002	0.0013	0.0004	0.0008	0.0001
	0.7672	0.7373	0.7619	0.7578	0.7831	0.7557	0.7885	0.7444	0.7232	0.6939	0.8074	0.7313	

	**NEFH**		**GRG1**		**NFM**		**NGF**		**aFGF**			
	w/o	with	w/o	with	w/o	with	w/o	with	w/o	with		

*Fitness*	0.0021	0.0007	0.0017	0.0002	0.0048	0.0015	0.0144	0.0037	0.0152	0.0068		
*Sensitivity*	0.7367	0.7178	0.8696	0.8039	0.7875	0.7378	0.7725	0.7574	0.7689	0.7433		

**Figure 8:**
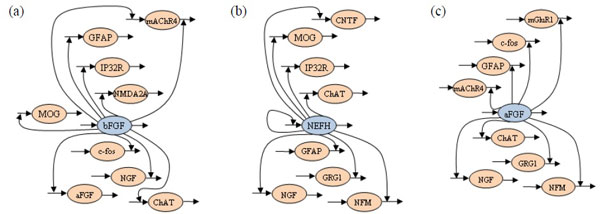
**The most sensitive regulations**. The most sensitive parameters (regulations) depicted by three pathway diagrams from the perspective of the (a) bFGF (b) NEFH, and (c) aFGF genes.

To further investigate the relationship between the gene roles and the sensitive network parameters identified by the proposed approach, we examined each sensitive parameter and tracked the genes that were related. The sensitive parameters described here are defined as the parameters that were recognized as sensitive in at least ten runs (from twenty runs). The results are listed in Table 6 in which the value indicates the number of sensitive parameters related to a specific gene. Three gene profiles, basic fibroblast growth factor (bFGF), neurofilament, heavy polypeptide (NEFH), and acidic fibroblast growth factor (aFGF), were involved in most of the gene regulations. According to this table, the two genes, bFGF and aFGF, dominated ten and eight gene regulations, respectively; in other words, the gene expression profiles that were being regulated were affected by the kinetic orders of bFGF and aFGF with the corresponding expression profiles. This finding indicates that the dynamics of bFGF and aFGF had a crucial influence on the network behavior of the inferred S-system model. In fact, bFGF and aFGF belong to the family of Fibroblast Growth Factors (FGFs), which has been shown to play an important role in gene duplications and gene repairs [33]. Similarly, the NEFH gene was related to nine gene regulations and also had a crucial influence on network reconstruction. The NEFH gene belongs to the gene group that controls key functions in nervous system development [34]; it provides instructions for making a protein component of neurofilaments. Figure 8 exhibits three pathway diagrams that illustrate the most crucial genetic interactions on the bFGF, NEFH, and aFGF genes. These observations show that our sensitivity analysis method can identify the most influential parameters of the biological pathways to be modeled. Using this method to select sensitive parameters and restrict them within certain ranges can efficiently improve the performance of the inference algorithms in the application of gene network reconstruction.

In the third set of experiments, we applied the proposed approach to a real dataset of yeast *S. cerevisiae *to infer the protein glycosylation system. Glycosylation plays an important role in biosynthesis activity; it is the enzymatic process attached to the target proteins that ensures that the proteins fold and assemble properly [35]. The associated expression data were first reported in [36], in which the authors began to obtain the synchronous yeast culture at the late G1 checkpoint. The cells were observed for nearly two full cell cycles, and 17 time points were collected (10 minutes per interval). As mentioned in the report, they reset the cell cycle at 110 minutes (i.e., the 12th time point); hence, we adopted the first complete cell cycle as the time-series dataset.

To form a regulatory network of the glycosylation system, we refer to the work by Spellman *et al*. [37], which has clustered 13 genes related to the glycosylation process. In addition, we exploited the software YeastNet version 2 ([38]) as our cluster checking tool to validate this cluster. YeastNet is a powerful theoretical framework that integrated several heterogeneous databases including encoding open reading frames (ORFs) of the yeast genome: *S. cerevisiae *Genome Database (SGD) [39]. In short, the software has calculated the confidences in pair-wise genetic interactions from the collected databases; thus, it provides a holistic view of yeast genes. Drawing on the calculation by YeastNet, there are 8 genes that remain in the glycosylation process; in other words, 5 genes have been deduced from the original cluster (with 13 genes), each of which has no connection to the others.

**Figure 9:**
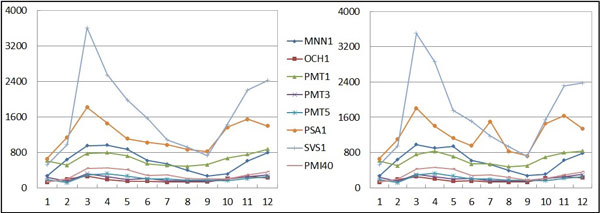
**Inferred network behavior**. (a) An overview of the inferred (left) and original (right) network behaviors of the yeast *S. cerevisiae *dataset.

In the experiments, similar to the previous sets of experiments, the hybrid GA-PSO method with and without sensitivity analysis has been employed to infer the glycosylation system of yeast *S. cerevisiae*. Table 8 compares the results, which show that the GA-PSO method with sensitivity analysis can deliver better performance on both the fitness and sensitivity results. Apart from the summary table, Figure 9 depicts the system behavior of the inferred and the original network. As observed, all of the profiles from the inferred model (left) are very similar to those of the target network (right), except for the PSA1 gene. This circumstance is mainly due to the measurement noise included in the PSA1 profile (from time point 6 to 8). According to the experimental discussion from Spellman *et al*. [37], the gene peaked only once in the G1 phase (i.e., between time points 1 and 4), and the second peak can be ignored. In other words, the inferred expression profile of PSA1 has captured the actual network behavior.

**Table 8:** Results of yeast *S. cerevisiae *dataset by different strategies.

	MNN1		OCH1		PMT1		PMT3	
	w/o	with	w/o	with	w/o	with	w/o	with
	
*fitness*	0.3486	0.1363	0.1999	0.0718	0.1501	0.0572	0.2929	0.1097
*sensitivity*	0.7750	0.7497	0.7975	0.7641	0.7770	0.7549	0.7833	0.7502
	**PMT5**		**PSA1**		**SVS1**		**PMI40**	
	w/o	with	w/o	with	w/o	with	w/o	with
	
*fitness*	0.1130	0.0862	0.3616	0.2692	0.7659	0.4456	0.4393	0.0676
*sensitivity*	0.7538	0.7464	0.8177	0.7846	0.7626	0.7428	0.8257	0.7495

**Figure 10:**
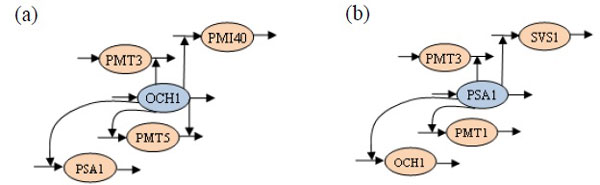
**The most crucial regulations**. The most crucial regulations are depicted in these two pathway diagrams, from the perspectives of the (a) OCH1 and (b) PSA1 genes.

Table 9 lists the number of sensitive parameters that are activated by each gene for further study. This table shows that there were two gene profiles, OCH1 (YGL038C) and PSA1 (YDL055C) genes, which dominated five and four gene regulations, respectively. Consequently, the network behavior was affected by the kinetic orders of OCH1 as well as PSA1 and their concentration levels. The OCH1 gene that catalyzes the chemical reaction encoding alpha-1,6-mannosyltransferase, is responsible for the outer chain initiation of *N*-linked oligosaccharides. It plays a key role in mannose elongation in *S. cerevisiae *[40]. On the other hand, the PSA1 gene, which is essential for cell wall integrity and morphogenesis, encodes GDP-mannose pyrophosphorylase (GDP-MP), in which the GDP-mannose is synthesized from Mannose-1-phosphate guanylyltransferase (GDP) and Guanosine-5'-triphosphate (GTP) through GDP-MP. This reaction is a critical step in synthesizing GDP-mannose for protein glycoproteins [41]. Figure 10 exhibits the most crucial genetic regulations in the inferred network by the OCH1 and PSA1 genes. These results again confirm the abilities and advantages of our integrated approach in network inference.

**Table 9:** The gene set and the number of sensitive parameters (regulations) activated by each gene in the synthesis or degradation process.

gene	OCH1	PSA1	PMI40	PMT5
*regulations*	5	4	3	1
gene	SVS1	MNN1	PMT1	PMT3
*regulations*	1	0	0	0

## Conclusions

In this study, we indicated the importance of considering both the network behavior and the network structure when inferring gene networks. We also developed an integrated approach that considers network robustness as an alternative to structural information in the inference procedure, when the relevant knowledge is not available. Based on the observation that the gene regulatory network was often dominated by only a small set of critical genes, our approach includes a parameter identification procedure that selects sensitive parameters and determines their value ranges. Our approach also includes a parameter optimization procedure that searches for the best fitting solutions. The above two procedures work iteratively, and in this way, the inferred networks can satisfy both requirements simultaneously.

A series of experiments have been conducted to validate the proposed approach. In our experiments, we investigated extensively analyzed the results that were obtained from three real gene datasets: the subsets from the SOS repair system, rat CNS, and yeast *S. cerevisiae*. The results confirm that the proposed approach can successfully identify and exploit the critical parameters that correspond to the genes that have active interactions in the biological systems. These genes dominate the network dynamics and therefore can enforce the inferred network structures to be close to the real target networks.

## Authors' contributions

YH undertook the experimental implementation, made statistical analysis and wrote a part of the manuscript. WL conceived the project, designed the algorithm, and wrote a part of the manuscript.

## Competing interests

The authors declare that they have no competing interests.
